# C5aR1 interacts with TLR2 in osteoblasts and stimulates the osteoclast‐inducing chemokine CXCL10

**DOI:** 10.1111/jcmm.13873

**Published:** 2018-09-24

**Authors:** Yvonne Mödinger, Anna Rapp, Julia Pazmandi, Anna Vikman, Karlheinz Holzmann, Melanie Haffner‐Luntzer, Markus Huber‐Lang, Anita Ignatius

**Affiliations:** ^1^ Institute of Orthopaedic Research and Biomechanics Trauma Research Center Ulm Ulm University Hospital Ulm Germany; ^2^ Ludwig Boltzmann Institute for Rare and Undiagnosed Diseases Vienna Austria; ^3^ Core Facility Genomics Ulm University Ulm Germany; ^4^ Institute of Clinical and Experimental Trauma‐Immunology University Hospital Ulm Ulm Germany

**Keywords:** bone inflammation, C5a, C5aR1, complement system, osteoblast, TLR2, toll‐like receptor

## Abstract

The anaphylatoxin C5a is generated upon activation of the complement system, a crucial arm of innate immunity. C5a mediates proinflammatory actions via the C5a receptor C5aR1 and thereby promotes host defence, but also modulates tissue homeostasis. There is evidence that the C5a/C5aR1 axis is critically involved both in physiological bone turnover and in inflammatory conditions affecting bone, including osteoarthritis, periodontitis, and bone fractures. C5a induces the migration and secretion of proinflammatory cytokines of osteoblasts. However, the underlying mechanisms remain elusive. Therefore, in this study we aimed to determine C5a‐mediated downstream signalling in osteoblasts. Using a whole‐genome microarray approach, we demonstrate that C5a activates mitogen‐activated protein kinases (MAPKs) and regulates the expression of genes involved in pathways related to insulin, transforming growth factor‐β and the activator protein‐1 transcription factor. Interestingly, using coimmunoprecipitation, we found an interaction between C5aR1 and Toll‐like receptor 2 (TLR2) in osteoblasts. The C5aR1‐ and TLR2‐signalling pathways converge on the activation of p38 MAPK and the generation of C‐X‐C motif chemokine 10, which functions, among others, as an osteoclastogenic factor. In conclusion, C5a‐stimulated osteoblasts might modulate osteoclast activity and contribute to immunomodulation in inflammatory bone disorders.

## 1 INTRODUCTION

The anaphylatoxin C5a is a potent mediator of innate immunity and promotes inflammation via its receptor C5aR1 upon activation of the complement system by exogenous and endogenous pathogen‐ and danger‐associated molecular patterns (PAMPs and DAMPs). Since the complement system can rapidly react on inflammatory stimuli, it can promote adequate host defence. Additionally, the complement system influences the development, homeostatic turnover, and regeneration of several tissues.^1^, ^2^ An increasing body of osteoimmunological research has revealed that bone tissue is subject to complement activation and modulation.^3^, ^4^ Bone tissue is continuously rebuilt during bone remodelling, a dynamic balance between bone‐forming osteoblasts and bone‐resorbing osteoclasts, which is essential for maintaining bone mass. Interestingly, osteoblasts strongly upregulate C5aR1 expression during differentiation, indicating an important function in these cells.^5^, ^6^ Indeed, in osteoblasts C5a induces migration and expression of the inflammatory cytokines interleukin‐6 (IL‐6) and IL‐8, and receptor activator of nuclear factor kappa B ligand (RANKL), which is essential for osteoclast formation and activity.^5^, ^7^ Moreover, C5aR1‐knockout (‐ko) mice display reduced osteoclast numbers and significantly increased bone mass, suggesting that C5a/C5aR1 signalling might regulate physiological bone turnover.^8^ The C5a/C5aR1 axis in bone cells might be particularly relevant under pathological conditions, because mice lacking C5aR1 are protected against arthritis,^9^ and C5aR1 activity has been linked to substantial bone loss in a periodontitis model.^10^ Antagonizing C5aR1 significantly reduced periodontal inflammation and subsequent bone loss in this model.^11^ Moreover, we previously demonstrated that C5aR1 was strongly expressed in osteoblasts in response to bone injury,^7^ and that bone fracture healing in a rodent model of severe systemic inflammation significantly improved when treated with a small peptide C5aR1 antagonist.^12^ In this setting, osteoblasts were found to be target cells for C5a, because mice with an osteoblast‐specific C5aR1 overexpression displayed impaired fracture healing.^6^

However, the molecular mechanisms underlying the C5a/C5aR1 signalling axis in osteoblasts remain unclear, also in respect of potential cross‐talking signalling pathways, which can modulate or are modulated by C5aR1 actions. In immune cells, C5aR1 has been described to interact with other immune receptors, including receptors for immunoglobulin G (IgG) antibodies, the FcγRs,^13^ or with other biological systems, including the coagulation cascade.^14^, ^15^ Toll‐like receptors (TLRs) are further potential interaction candidates, because, similar to the complement system and its receptors, they are important for early recognition and adequate response to danger molecules.^16^ In this regard, pathways downstream of complement receptors and TLRs interact in various immune cells,^17^, ^18^ thereby modulating inflammatory responses.^19^ In this study, we aimed to determine the intracellular events following C5aR1 activation in osteoblasts. We analysed gene expression patterns and intracellular signalling pathways upon C5aR1 activation and found a strong modulation of genes involved for example in the mitogen‐activated protein kinase (MAPK) and insulin pathways. Furthermore, we demonstrated that C5aR1 and TLR2 interact in osteoblasts, resulting in upregulation of the immune cell chemoattractant C‐X‐C motif chemokine 10 (CXCL10), which can induce osteoclastic bone resorption.^20^, ^21^, ^22^ These results suggest that complement‐activated osteoblasts are able to modulate the inflammatory milieu during inflammatory bone diseases in concert with osteoclasts and immune cells.

## 2 MATERIALS AND METHODS

### 2.1 Mouse model

Male wild‐type (WT) control (C57BL/6) mice were purchased from Charles River (Sulzfeld, Germany) while C5aR1‐ko mice, originally generated by C. Gerard 23 and kindly provided by John D. Lambris (University of Pennsylvania, USA), were bred in‐house. Mice were housed according to the guidelines for the care and use of laboratory animals (ARRIVE) and had access to a standard mouse feed (ssniffR R/M‐H, V1535‐300, Ssniff, Soest, Germany) and water ad libitum. Experiments were performed with permission of the local authorities.

### 2.2 Osteoblast isolation, cultivation, and stimulation

Primary osteoblasts were isolated from long bones of 8–12‐week‐old mice and differentiated for 14 days, as described previously.^6^, ^24^ Briefly, harvested diaphyses were shred and digested for 2 h using 125 U/ml collagenase type II (Sigma‐Aldrich, Steinheim, Germany) in modified Minimal Essential Medium (α‐MEM, Biochrom, Berlin, Germany). For osteoblast outgrowth, bone chips were placed into α‐MEM supplemented with 10% heat‐inactivated foetal calf serum (FCS), 100 U/ml penicillin/streptomycin, 1% L‐glutamine and 0.5% Fungizone^TM^ (amphotericin B) (all from Gibco, Darmstadt, Germany) at 37°C under 5% CO_2_. Passage 2 osteoblasts and passage 7 MC3T3‐E1 cells (ATCC^®^ CRL‐2593) were used for the experiments. Osteogenic differentiation was induced by adding 0.2 mM ascorbate‐2‐phosphate and 10 mM β‐glycerophosphate (both Sigma‐Aldrich). Differentiated osteoblasts were stimulated using 100 ng/ml murine recombinant C5a (Hycultec GmbH, Beutelsbach, Germany), 100 ng/ml Pam3CSK4 (Pam3) (TLR2‐agonist, Invivogen, San Diego, USA), or both, for 10 min, 30 min, 1 h, 2 h, 4 h, 6 h, or 24 h. Pretreatment of osteoblasts with C5aR1 antagonist PMX‐53 (1.1 μg/ml, kindly supplied by John D. Lambris), p38 MAPK inhibitor SB 203580 (10 μM, Calbiochem, Merck KGaA, Darmstadt, Germany) or myeloid differentiation primary response 88 (MyD88) inhibitor ST 2825 (10 μM, Hycultec GmbH) was performed for 1 h before adding C5a and/or Pam3 for the indicated time periods.

### 2.3 Osteoclast formation and activity assay upon stimulation with osteoblast supernatant

Cell‐culture supernatants of osteoblasts, left unstimulated or stimulated for 4 h with C5a and/or Pam3, were applied on osteoclast cultures, to investigate the osteoclastogenic potential. RAW 264.7 (ATCC^®^ TIB‐71) cells were seeded at a density of 1500 cells/cm^2^ in 96‐well plates and differentiated into osteoclast‐like cells. Osteoblast supernatants were added to the osteoclast medium (DMEM (ATCC, Manassas, USA), supplemented with 10% heat‐inactivated FCS, 1% penicillin/streptomycin and 1% L‐glutamine (all from Gibco)) in a 1:1 ratio and with a final concentration of 10 ng/ml RANKL (462‐TEC, R&D Systems, Wiesbaden, Germany) and 5 ng/ml human recombinant M‐CSF (Merck). Additionally, PMX‐53 (1.1 μg/ml), MyD88 inhibitor ST 2825 (10 μM), and a CXCL10‐antibody (10 μg/ml, R&D Systems) were applied together with the osteoblast supernatant. In addition, control groups were included, where C5a (100 ng/ml), Pam3 (100 ng/ml) or recombinant mouse CXCL10 (100 ng/ml, PeproTech, Hamburg, Germany), with or without CXCL10‐antibody, were added to the osteoclast medium without osteoblast supernatant. Cells were kept at 37°C under 5% CO_2_. Osteoclastogenic differentiation was assessed after 5 days by counting multinucleated (>2 nuclei) tartrate‐resistant acid phosphatase (TRAP)‐positive cells using light microscopy. Additionally, mRNA samples were obtained.

### 2.4 Enzyme‐linked immunosorbent assay (ELISA)

Cell‐culture supernatants of osteoblasts stimulated for 4 h with C5a and/or Pam3, were analysed according to the manufacturer's instructions using a mouse ELISA‐kit for CXCL10 (CRG‐2) (#EMCXCL10, Thermo Fisher Scientific). Data were analysed using a standard curve provided with the kit. Values below assay detection limit were set to zero.

### 2.5 Reverse transcription‐PCR (RT‐PCR)

Total RNA isolation and RT‐PCR were performed as described previously.^6^ Gene expression was analysed relative to the housekeeping gene glyceraldehyde‐3‐phosphate dehydrogenase (*Gapdh*) using the ΔΔCt method. Primers were purchased from Invitrogen (Thermo Fisher Scientific, Waltham, USA) and sequences are available in Table S1. Capillary gel electrophoresis and quantification of PCR products were performed using QIAxcel DNA Screening Gel Cartridge on a QIAxcel Advanced System (Qiagen, Hilden, Germany).

### 2.6 Microarray‐based gene expression analysis

Differentiated osteoblasts were untreated or stimulated with 100 ng/ml C5a for 30 min or 4 h (n = 3 per group). RNA was isolated and the RNA integrity number (RIN) determined using an Agilent Bioanalyser (Agilent Technologies, Santa Clara, USA). Samples with a RIN of ≥ 9.1 were used. Microarray analyses were performed using 200 ng total RNA and 5.5 μg single‐stranded DNA (ssDNA) per hybridization in a GeneChip^®^ Fluidics Station 450 (Affymetrix, Santa Clara, USA). Following RNA amplification and labelling, ssDNA was hybridized to Mouse Gene 1.0 ST GeneChip^®^ Arrays and scanned using a GeneChip^®^ scanner 3000 (both Affymetrix). Images were analysed using Affymetrix^®^ Expression Console Software and BRB‐ArrayTools (http://linus.nci.nih.gov/BRB-ArrayTools.html). Raw feature data were normalized and log2 intensity expression summary values were calculated using the robust multiarray average. Differentially expressed probesets were determined by *t* test and considered statistically significant when *P* < 0.05 and fold change ≤ 1.5, as published previously.^25^ Functional protein‐association networks were identified using the STRING 10 program (26 http://string-db.org/). Differentially expressed genes were subjected to pairwise gene ontology (GO) term similarity measure with Lin's algorithm using GOSemSim in R.^27^ Similarity matrices served as inputs for hierarchical clustering using the R package hclust. Enrichment analysis of the resulting groups was performed using EnrichR (28 http://amp.pharm.mssm.edu/Enrichr/). The GoMINER tool^29^ was used to identify the most affected biological processes and pathway analysis was performed using Transcriptome Analysis Console (Affymetrix). Complete microarray data are available at Gene Expression Omnibus (GEO accession number: GSE107036).

### 2.7 Coimmunoprecipitation

Immunoprecipitation of C5aR1 and subsequent C5aR1 and TLR2 detection by immunoblotting was performed using MC3T3‐E1 cells, untreated or stimulated for 1 h with 100 ng/ml C5a or Pam3. Cells were washed with ice‐cold phosphate‐buffered saline (PBS), and 1 mM DSP (dithiobis(succinimidyl propionate), Thermo Fisher Scientific) protein crosslinker was added for 30 min at room temperature (RT). The reaction was terminated by adding 1 mM Tris for 15 min at RT and the cells were lysed using 1 ml Pierce^®^ IP‐lysis buffer (Thermo Fisher Scientific), supplemented with 1 μl protease and phosphatase inhibitors (PPi) (Halt^TM^, Thermo Fisher Scientific). Cell lysates were precleared by incubation with rabbit serum for 1 h on ice before adding 100 μl protein‐A sepharose (EZview^TM^ Red Protein A Affinity Gel, Sigma‐Aldrich) for 30 min at 4°C under rotation. After centrifugation for 30 min at 14000 × g at 4°C, cell lysates were collected and incubated with beads, precoupled to 7.5 μg rabbit C5aR1 antibody (#APO6508PU‐N, Acris, Herford, Germany) or rabbit IgG antibody (#011‐000‐003, Jackson ImmunoResearch, West Grove, USA). Incubation was performed overnight at 4°C under rotation. Beads were washed with PTO buffer (pH 7.2, 20 mM sodium dihydrogenphosphate, 0.5% Tween 20, 0.1% albumin from chicken egg white (A5503), Sigma‐Aldrich). Protein complexes were detached from beads with 30 μl sample buffer (175 mM Tris (Merck), 5% sodium dodecyl sulphate (SDS), 15% glycerin, and 1% dithiothreitol (all Sigma‐Aldrich)) and incubating for 30 min at 37°C and 5 min at 95°C.

### 2.8 Immunoblotting

Cells were lysed in Pierce^®^ RIPA buffer (Thermo Fisher Scientific), containing PPi. Sample buffer was added to the protein samples and 10 μg total protein was resolved on a 10% SDS polyacrylamide gel in 25 mM Tris buffer, containing 0.1% SDS and 192 mM glycine (BioFroxx, Einhausen, Germany). Proteins were transferred to a nitrocellulose membrane (Amersham^TM^ Protran^TM^ 0.2 m NC, GE Healthcare, Chicago, USA) in 25 mM Tris buffer, containing 192 mM glycine and 20% methanol for 1 h at 80 V. Tris‐buffered saline with 1% Tween 20 and 3% bovine serum albumin was used for blocking. Primary rat C5aR1 (CD88) (#MA1‐81761, 1:1000, Thermo Fisher Scientific), rabbit p38 (#8690S, 1:1000, Cell Signalling Technology, Danvers, USA), rabbit p‐p38 (#4511S, 1:1000, Cell Signalling Technology), rabbit TLR2 (#bs‐1019R, 1:1000, Bioss, Woburn, USA), and rabbit GAPDH antibodies (#14C10, 1:1000, Cell Signalling Technology) were incubated for 12 h at RT, followed by a secondary horseradish peroxidase (HRP)‐linked anti‐rat (#61‐9520, 1:20.000, Zymed Laboratories, Inc., Thermo Fisher Scientific) or anti‐rabbit antibody (#7074P2, 1:15.000, Cell Signalling Technology) for 2 h at RT. WesternBright^TM^ ECL chemiluminescent HRP substrate (Advansta, Menlo Park, USA) was applied to the membranes for 2 min at RT and the signal was captured by membrane exposure to X‐ray film (CL‐XPosure^TM^, Thermo Fisher Scientific). Western blot images were quantified by use of densitometry values derived by Adobe Photoshop CS6 and are presented relative to GAPDH.

### 2.9 Immunofluorescent staining

Osteoblasts were fixed in 4% phosphate‐buffered formalin and unspecific binding was prevented using goat serum for 1 h at RT. Cells were permeabilized with 0.1% Triton X‐100 before incubation with rabbit C5aR1 antibody (#ab59390, 1:100, Abcam, Cambridge, UK), rabbit TLR2 antibody (#bs‐1019R, 1:100, Bioss) or rabbit IgG antibody (#sc‐2027, 1:100, Santa Cruz Biotechnology, Dallas, USA) overnight at 4°C. Cells were washed with PBS and incubated with a goat anti‐rabbit biotinylated antibody (#B2770, 1:100, Life Technologies) for 1 h at RT, followed by streptavidin‐FITC (#405202, 1:100, BioLegend, San Diego, USA) for 1 h at RT. Counterstaining of nuclei was performed with Hoechst 33258 (Sigma‐Aldrich). Images were acquired using a DMI6000B microscope (Leica, Wetzlar, Germany) and the LASX software (v. 2.0.0.14332, Leica).

### 2.10 Statistical analysis

Results are presented as the mean ± standard deviation. For statistical analysis, the software GraphPad Prism 6 (GraphPad Software, Inc., La Jolla, USA) was used. Testing for normal distribution was performed using the Shapiro‐Wilk test. Student's *t* test was applied when two groups were compared while one‐way analysis of variance (ANOVA), followed by Fisher's LSD post hoc test, was applied to compare three or more groups. The level of significance was set at *P* ≤ 0.05.

## 3 Results

### 3.1 C5a regulates genes involved in the MAPK‐ and transforming growth factor (TGF)‐β pathways, insulin signalling, and the activator protein (AP)‐1 transcription factor in osteoblasts

**Figure 1:**
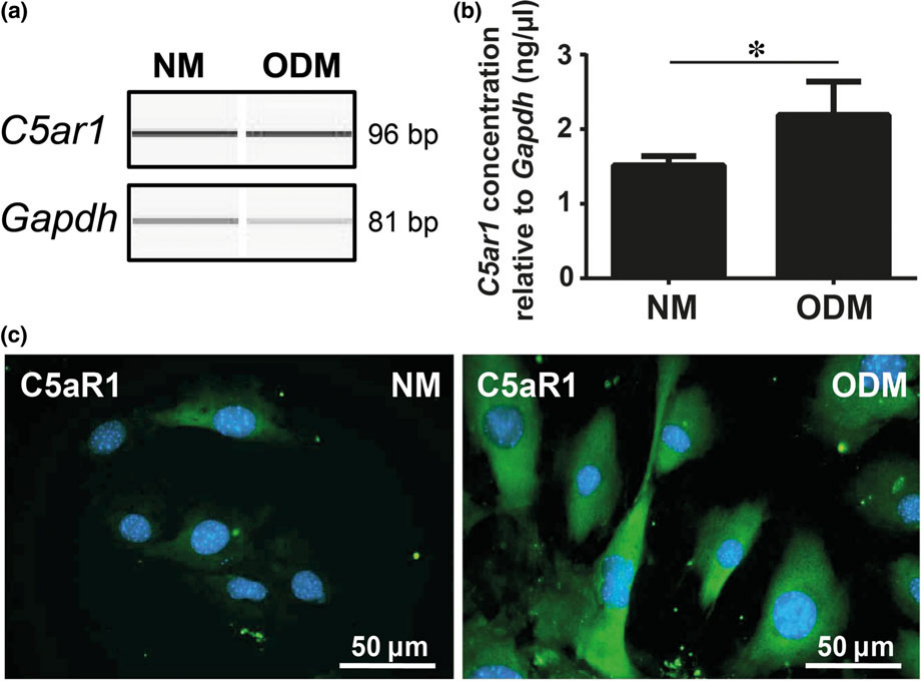
C5aR1 gene and protein expression in immature and mature osteoblasts. C5ar1 gene expression in immature (NM) and mature osteoblasts (ODM) (A). Gapdh serves as a reference and images were obtained by capillary electrophoresis. Concentration of C5ar1 relative to Gapdh in immature (NM) and mature osteoblasts (ODM) (B). Immunofluorescent staining of C5aR1 in immature (NM) and mature osteoblasts (ODM) in green. Nuclei are stained in blue (C). NM: normal proliferation medium, ODM: osteogenic differentiation medium. *P ≤ 0.05, n = 5

**Figure 2:**
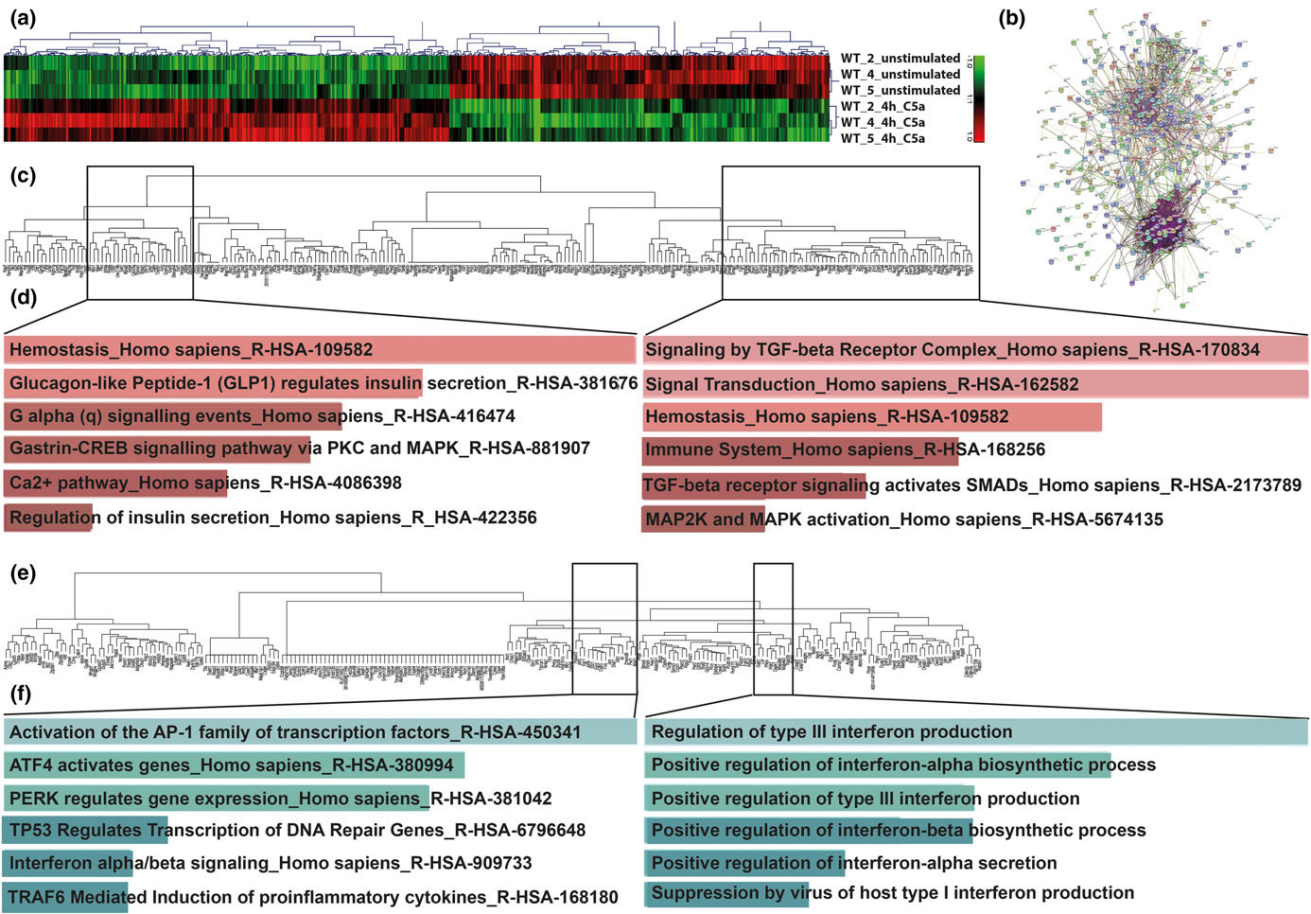
Gene expression profiling of osteoblasts stimulated with C5a for 4 h. Representative heat‐map segment derived from hierarchical clustering of differentially regulated genes after 4 h‐stimulation with C5a, using Pearson uncentred as similarity measure and average linkage clustering (A). Nondetailed STRING protein‐association network of differentially regulated genes after 4‐h stimulation with C5a (B). Dendrogram of upregulated genes upon C5a treatment, obtained by hierarchical clustering using the gene ontology (GO) category Molecular Function (C). Molecular function of the enriched genes from the in (C) depicted representative gene clusters (D). Dendrogram of downregulated genes upon C5a treatment, obtained by hierarchical clustering using the GO category Molecular Function (E). Molecular function and biological pathway involvement of the enriched genes from the in (E) depicted representative gene clusters (F). N = 3 per group (unstimulated and stimulated)

Confirming previous findings,^5^, ^6^ increased *C5ar1* expression levels were detected in osteoblasts cultured in osteogenic differentiation medium compared to cells in normal proliferation medium (Figure 1A and B). Immunofluorescent staining demonstrated strong C5aR1 upregulation upon differentiation (Figure 1C). To investigate actions conveyed by the C5a/C5aR1 axis, we performed gene expression profiling of C5a‐stimulated osteoblasts. In total, 606 probesets were differentially regulated, which strongly clustered between the treatment groups, as visualized by heat‐map (Figure 2A), and regarding their presumed protein functions, exemplified by the protein‐association network (Figure 2B). After excluding probesets not assigned to an Entrez Gene ID, 472 probesets remained for further analysis, whereof 241 probesets were upregulated (Figure 2C) and 231 probesets were downregulated (Figure 2E). Hierarchical clustering and enrichment analysis revealed that upregulated genes are involved in insulin signalling and secretion, TGF‐β receptor signalling and the activation of MAPKs (Figure 2D). Downregulated genes are involved in AP‐1 transcription factor formation and interferon production and signalling (Figure 2F). By performing pathway analysis of all regulated genes, insulin metabolism, TGF‐β signalling, MAPK regulation and interferon signalling appeared among the top C5a‐regulated biological pathways (Table 1), thus confirming data derived from the enrichment analyses (Figure 2D and F). A list of the top 20 regulated pathways upon C5a stimulation is provided in the supplement (Table S2). To confirm microarray findings, differential expression of selected candidate genes was validated by RT‐PCR. Regarding insulin and glucose metabolism, we confirmed the upregulation of glutamine fructose 6‐phosphate transaminase 2 (*Gfpt2*), an enzyme regulating glucose metabolism. Genes encoding negative regulators of insulin signalling were downregulated, including suppressor of cytokine signalling 3 (*Socs3)* and growth factor receptor‐bound protein 14 (*Grb14*), an important insulin receptor adaptor protein (Table 2). Therefore, glucose‐ and insulin‐related signalling in osteoblasts appears to be regulated by C5a. Genes involved in the TGF‐β pathway, namely TGF‐β receptor 1 (*Tgfbr1*), TGF‐β‐induced protein (*Tgfbi*), TGF‐β‐induced factor homeobox 1 (*Tgif1*), and TGF‐β (*Tgfb1*) itself were induced by C5a (Table 2). We further confirmed that MAPK6 (*Mapk6*) and MAPK kinase 3 (*Map2k3*) were slightly upregulated, whereas negative MAPK regulators dual specificity phosphatase 1 (*Dusp1*) and *Dusp5* were downregulated (Table 2). This shows that C5a activates MAPKsignalling in osteoblasts. The AP‐1 transcription factor subunits *Fos* and *Jun* (Table 2), and levels of other immediate early genes, including immediate early response 2 and 3 (*Ier2*,* Ier3*) and early growth response 2 and 3 (*Egr2, Egr3*) (Table S3) were considerably reduced. There was a high correlation (R^2^ = 0.957) between the logarithmic fold change values derived from the microarray and RT‐PCR analyses (Figure S2). Complete lists of the top 20 upregulated (Table S3) and downregulated genes (Table S4) upon 4 h‐C5a‐treatment are provided in the supplement.

**Table 1:** Top regulated pathways upon C5a treatment in osteoblasts

Order	Pathway	Genes up	Genes down	Significance	*P*‐value
1	Insulin signalling	8	12	9.48	<1 × 10^–6^
2	TGF‐beta receptor signalling	9	8	7.47	<1 × 10^–6^
3	TGF‐beta signalling	6	4	6.80	<1 × 10^–6^
4	MAPK signalling	6	10	6.39	<1 × 10^–6^
5	MAPK signalling	6	10	5.99	1 × 10^–6^
10	Type II interferon signalling	0	7	5.14	7 × 10^–6^
11	p38 MAPK signalling	2	4	4.08	8 × 10^–5^
16	Toll‐like receptor signalling	2	6	2.89	1.3 × 10^–2^
25	MAPK cascade	2	2	2.46	3.5 × 10^–2^

The main regulated biological pathways, determined by using the Transcriptome Analysis Console Software, are shown in descending order, based on their significance. The number of up‐ and downregulated genes, included in the respective pathways according to the gene ontology term, is shown in columns 3 and 4. The *P*‐value of differential regulation and its negative decadal logarithm (significance) are shown in column 6 and 5, respectively. A complete list of the top 20 regulated pathways is provided in the supplement (Table S1).

**Table 2:** Selected differentially regulated genes upon C5a treatment in osteoblasts

Gene	Description	GenBank^a^	*P*‐value	FC MA^b^	FC PCR^c^
*Fos*	FBJ osteosarcoma oncogene	NM_010234	1 × 10^–5^	28.6 ↓	109.1 ↓
*Fosb*	FBJ osteosarcoma oncogene B	NM_008036	5.2 × 10^–3^	12.4 ↓	NA
*Dusp1*	Dual specificity phosphatase 1	NM_013642	1.9 × 10^–2^	4.4 ↓	15.0 ↓
*Junb*	Jun B proto‐oncogene	NM_008416	2.4 × 10^–3^	4.0 ↓	NA
*Dusp5*	Dual specificity phosphatase 5	NM_001085390	8.4 × 10^–3^	3.9 ↓	6.8 ↓
*Jun*	Jun proto‐oncogene	NM_010591	7.3 × 10^–3^	2.9 ↓	6.2 ↓
*Grb14*	GF receptor‐bound protein 14	NM_016719	4.9 × 10^–2^	2.0 ↓	2.8 ↓
*Socs3*	Suppressor of cytokine signalling 3	NM_007707	7.8 × 10^–3^	1.9 ↓	4.2 ↓
*Mapk6*	MAPK 6	NM_015806	4.8 × 10^–2^	1.6 ↑	2.1 ↑
*Tgif1*	TGF‐beta‐induced factor homeobox1	NM_001164074	4 × 10^–2^	1.6 ↑	NA
*Map2k3*	MAPK kinase 3	NM_008928	2.6 × 10^–2^	1.7 ↑	1.3 ↑
*Tgfbr1*	TGF‐beta receptor 1	NM_009370	2.1 × 10^–2^	2.1 ↑	1.5 ↑
*Tgfb1*	TGF‐beta	NM_011577		ND	1.8 ↑
*Gfpt2*	Glutamine F‐6‐P transaminase 2	NM_013529	1.9 × 10^–3^	4.3 ↑	5.1 ↑
*Tgfbi*	TGF‐beta induced	NM_009369	3.8 × 10^–3^	4.9 ↑	2.5 ↑

Selected regulated probesets upon C5a exposure, representing respective genes, are shown in descending order for the downregulated and in ascending order for the upregulated genes. The respective *P*‐value of differential regulation and the fold change compared to unstimulated osteoblasts, as derived from microarray analysis, are depicted in columns 4‐6, as well as the respective gene accession number (column 3). Complete lists of the top 20 up‐ and downregulated genes are provided in the supplement (Table S3 and S4, respectively). GF: growth factor, MAPK: mitogen‐activated protein kinase, F‐6‐P: fructose‐6‐phosphate, TGF‐beta: transforming growth factor‐beta.

a GenBank Accession Number.

b fold change (FC), derived from microarray analysis.

c FC derived from RT‐PCR, ↑positive FC, ↓negative FC. NA: not assessed. ND: not detected.

### 3.2 C5aR1 and TLR2 interact in osteoblasts and downstream signalling involves the activation of p38 MAPK

**Figure 3:**
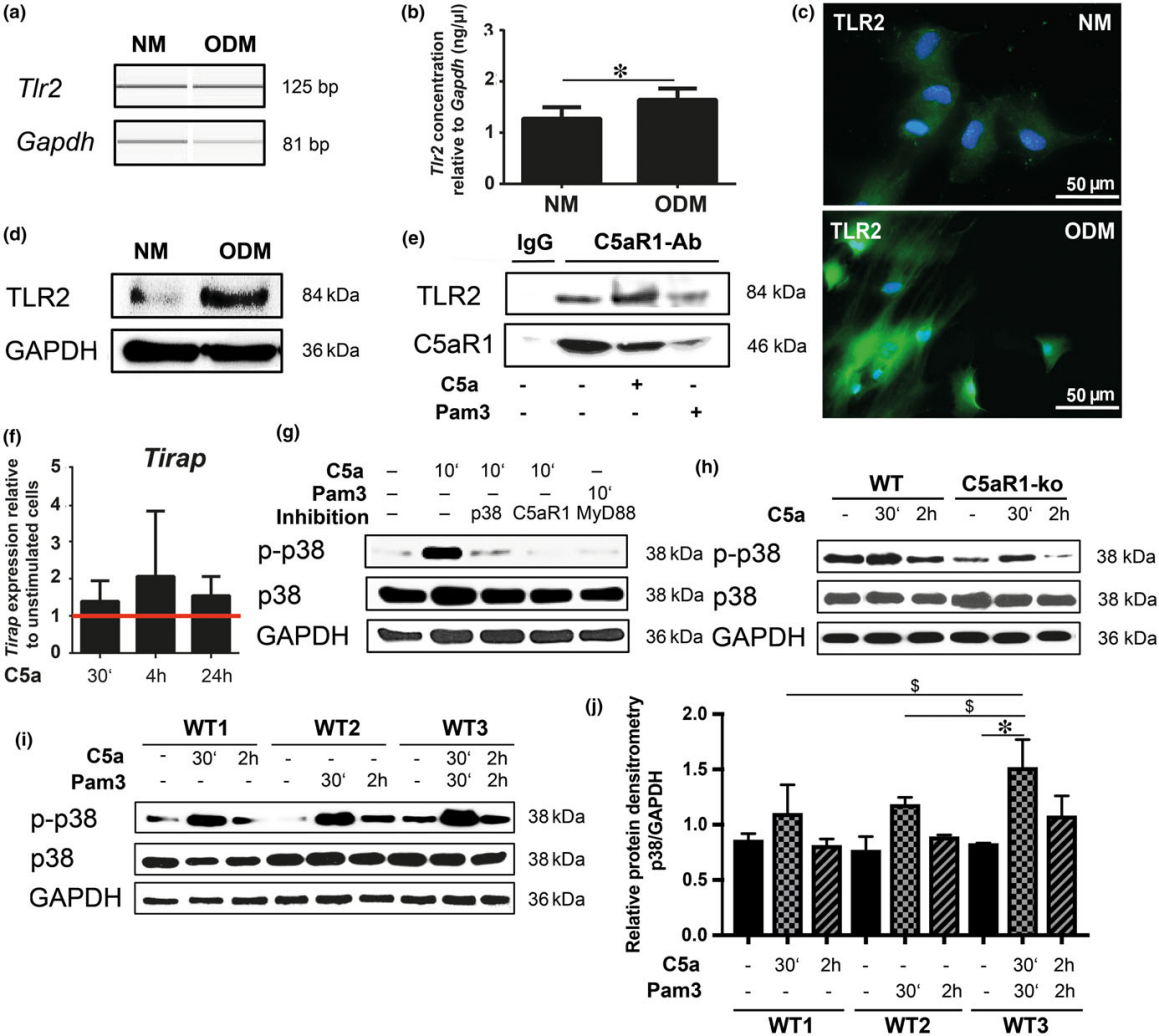
C5aR1 and TLR2 interact and activate p38 MAPK in osteoblasts. Expression of TLR2 in immature (NM) and mature osteoblasts (ODM), on gene level (A), quantified relative to Gapdh (B). Images in A were obtained by using capillary electrophoresis. TLR2 protein expression in immature (NM) and mature (ODM) osteoblasts, shown by immunofluorescent staining in green, while nuclei are counterstained in blue (C) and shown by immunoblotting (D). C5aR1 and TLR2 immunoblotting of protein lysates, immunoprecipitated using a C5aR1 or IgG antibody (E). Tirap gene expression after stimulation with C5a for 30 min, 4 h or 24 h. The red line indicates the relative gene expression of unstimulated cells set to 1 (F). Immunoblotting for p38/p‐p38 using WT untreated osteoblasts or treated with C5a or Pam3 for 10 min, with or without inhibition of p38, C5aR1 or MyD88, using SB 203580, PMX‐53 and ST 2825, respectively (G). Immunoblotting for p38/p‐p38 using WT and C5aR1‐ko osteoblasts left untreated or treated with C5a for 30 min or 2 h (H). Immunoblotting for p38/p‐p38 using wild‐type (WT) osteoblasts left untreated or treated with C5a (lanes 2, 3), Pam3 (lanes 5, 6), or both (lanes 8, 9) for 30 min or 2 h (I). Western blot protein band quantification by densitometry, shown as ratio between p38 and GAPDH. $ *P* ≤ 0.05 between the different 30 min‐treatment groups, * *P* ≤ 0.05 between the unstimulated and 30 min treatment sample of the same treatment group (J). N = 4–6 per treatment (A, B, E). GAPDH serves as a loading control (B, F–H). P‐p38: phosphorylated p38 MAPK protein, Pam3: Pam3CSK4, GAPDH: glyceraldehyde‐3‐phosphate dehydrogenase, Tirap: Toll‐interleukin 1 receptor (TIR) domain‐containing adaptor protein. MyD88: myeloid differentiation primary response 88, NM: normal proliferation medium, ODM: osteogenic differentiation medium

‘Toll‐like Receptor Signalling’ was among the top C5a‐regulated pathways (Table 1) and thus any interplay between C5aR1 and TLR2 in osteoblasts was of special interest. We first analysed TLR2 expression and detected slightly but significantly upregulated gene levels upon osteogenic differentiation (Figure 3A and B). TLR2 protein expression was greatly enhanced upon differentiation, as shown by both immunofluorescence (Figure 3C) and immunoblotting (Figure 3D). Coimmunoprecipitation of C5aR1 was performed to analyse the presence of C5aR1‐TLR2 complexes in osteoblasts, with and without prestimulation of the receptors with their specific ligands C5a and Pam3. Immunoblotting showed that TLR2 coimmunoprecipitated with C5aR1, demonstrating a physical interaction in osteoblasts (Figure 3E). This interaction was apparent already under unstimulated conditions, and increased when prestimulating C5aR1. Stimulation of TLR2 with Pam3 did not further increase receptor interactions (Figure 3E). We did not find evidence supporting a reciprocal regulation of the receptors, as C5aR1 gene and protein expression was unaltered after stimulation with Pam3, neither were levels of TLR2 altered upon stimulation with C5a (Figure S2). Nevertheless, we confirmed microarray findings, that C5a treatment enhanced levels of Toll‐interleukin 1 receptor domain‐containing adaptor protein (*Tirap*) (Figure 3F), an important molecule for TLR downstream actions. *Tirap* levels were increased as early as 30 min and remained high until 24 h after C5a stimulation. Therefore, C5a appears to enhance TLR2 downstream signalling rapidly and persistently (Figure 3F). For confirmation, we investigated receptor‐mediated activation of MAPKs, which are intracellular signalling transducers, and which we found being regulated by C5a on gene level (Figure 2D; Table 2). MAPKs p38 and ERK1/2 (data not shown) were phosphorylated and thereby activated by C5a (Figure 3I). The same effect was observed after TLR2 stimulation. Notably, p38 phosphorylation significantly increased more at 30 min after receptor costimulation, compared to isolated receptor stimulation (Figure 3I), which was confirmed by the quantification of the western blot protein bands (Figure 3J). This finding suggests combined actions of C5aR1 and TLR2 in downstream signalling. Importantly, inhibition of C5aR1 with its antagonist PMX‐53 prevented p38 phosphorylation (Figure 3G). In line, p38 phosphorylation was considerably diminished in C5aR1‐ko osteoblasts (Figure 3H), corroborating C5aR1‐dependent MAPK activation. Additionally, TLR2‐mediated p38 phosphorylation was prevented by inhibiting the TLR adaptor protein MyD88 (Figure 3G). Therefore, in addition to C5aR1, signalling via p38 MAPK is also TLR2‐dependent.

### 3.3 C5aR1 and TLR2 promote upregulation and secretion of CXCL10 in osteoblasts via p38 MAPK

**Figure 4:**
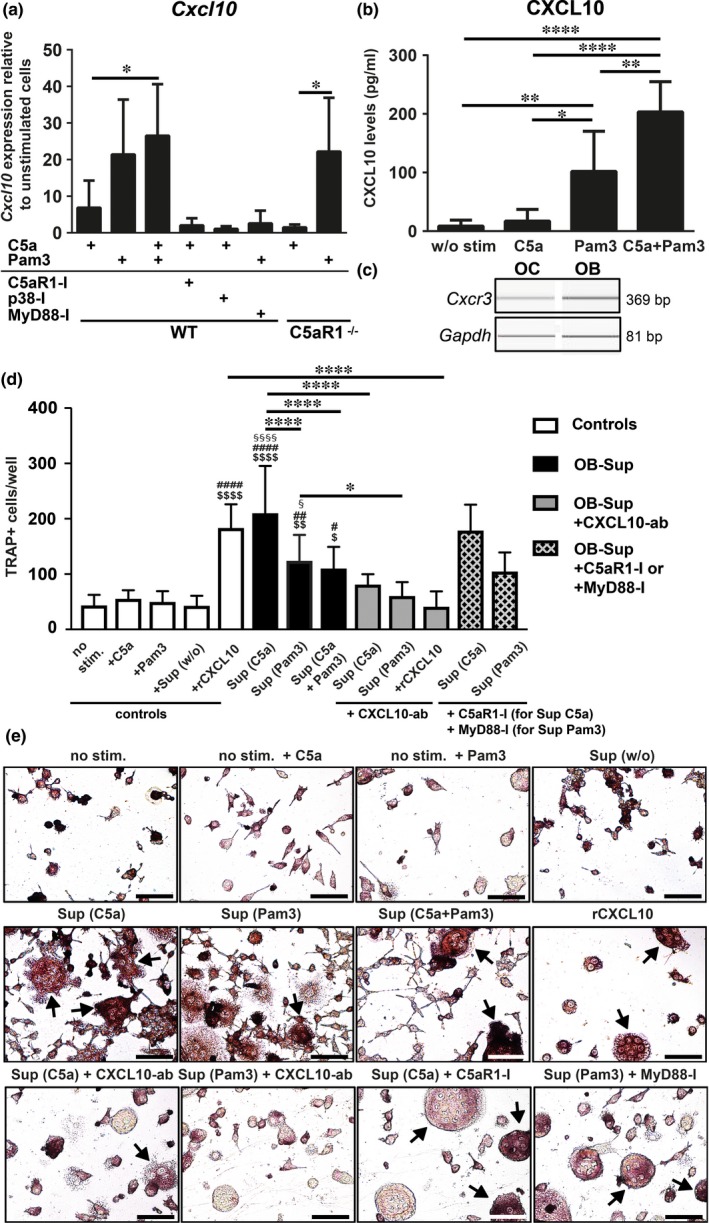
C5aR1 and TLR2 promote p38 MAPK‐dependent upregulation and secretion of CXCL10 in osteoblasts, inducing osteoclast formation. Cxcl10 expression in wild‐type (WT) and C5aR1‐ko osteoblasts upon treatment with C5a and/or Pam3 for 4 h, with or without pharmacological inhibition of C5aR1, p38 or MyD88, using PMX‐53, SB 203580 and ST 2825, respectively (A). * *P* ≤ 0.05, in respect to C5a‐treated WT or C5aR1‐ko cells, n = 3‐5 (A). CXCL10 protein levels in cell‐culture supernatants of osteoblasts left unstimulated (w/o stim) or stimulated with C5a and/or Pam3 for 4 h (B). * *P* ≤ 0.05, ** *P* ≤ 0.01, **** *P* ≤ 0.0001, n = 3‐5 (B). Cxcr3 gene expression in differentiated osteoblasts and osteoclasts. Gapdh serves as a reference and images were obtained using capillary electrophoresis (C). Quantification of TRAP‐stained RAW 264.7 osteoclast‐like cells, which where incubated with or without osteoblast supernatant, rCXCL10, C5a or Pam3. Additional groups underwent inhibition of C5aR1, MyD88 or CXCL10, as indicated. (D). $ *P* ≤ 0.05, $$ *P* ≤ 0.01, $$$$ *P* ≤ 0.0001, in respect to untreated cells (no stim.). # *P* ≤ 0.05, ## *P* ≤ 0.01, #### *P* ≤ 0.0001, in respect to cells receiving untreated osteoblast supernatant (Sup (w/o)). §§§ *P* ≤ 0.001, §§§§ *P* ≤ 0.0001, in respect to cells receiving C5a or Pam3. * *P* ≤ 0.05, ****, *P* ≤ 0.0001 between groups, n = 4‐8 (D). Images of TRAP‐stained RAW 264.7 osteoclast‐like cells, which were identified by increased size, multiple nuclei, and TRAP‐positive red staining (exemplified by arrows). Respective image of cells treated with recombinant CXCL10 and CXCL10‐ab is not shown. Scale bar: 50 μm (E). Pam3: Pam3CSK4, MyD88: myeloid differentiation primary response 88, rCXCL10: recombinant C‐X‐C motif chemokine 10, CXCR3: C‐X‐C motif chemokine receptor 3, OB: osteoblasts, OC: osteoclasts, TRAP: Tartrate‐resistant acid phosphatase, Sup: osteoblast cell‐culture supernatant, w/o: without, ab: antibody

Having demonstrated that C5aR1 and TLR2 induce p38 activation individually and in combination, we investigated potential downstream targets and focused on the inflammatory response of osteoblasts. Herein, we found CXCL10 gene (*Cxcl10*) levels to be strongly upregulated after 4 h of C5a exposure compared to unstimulated cells (Figure 4A). Notably, *Cxcl10* was already upregulated after 30 min of C5a treatment in both microarray and RT‐PCR (data not shown). TLR2 stimulation led to an even greater *Cxcl10* increase, and, similar to the effect on p38, *Cxcl10* levels were further and significantly enhanced after receptor costimulation (Figure 4A), suggesting additive actions of the underlying pathways. Importantly, C5a did not enhance *Cxcl10* when inhibiting C5aR1 or p38 MAPK pharmacologically (Figure 4A), indicating a receptor‐ and p38 MAPK‐dependent induction. Additionally, Pam3‐mediated *Cxcl10* induction was prevented when inhibiting MyD88 pharmacologically (Figure 4A). Furthermore, *Cxcl10* was not induced when exposing C5aR1‐ko osteoblasts to C5a, while Pam3 stimulation led to *Cxcl10* induction, similarly to Pam3‐stimulated WT osteoblasts (Figure 4A). In addition to gene expression, C5a marginally induced osteoblast secretion of CXCL10 into the cell‐culture supernatant (Figure 4B). This effect was however significantly stronger by Pam3 treatment, and again receptor costimulation led to significantly higher CXCL10 levels compared to isolated receptor stimulation (Figure 4B). C‐X‐C motif chemokine receptor 3 (CXCR3), which is the receptor for CXCL10, was found to be expressed by both osteoblasts and osteoclasts (Figure 4C). To analyse the osteoclastogenic potential of osteoblast‐secreted components, osteoblast cell‐culture supernatants were added to osteoclast precursor cells. The addition of supernatant following C5a and Pam3 treatment induced osteoclast formation in comparison to unstimulated controls ($) and to controls receiving untreated supernatant (#), as assessed by TRAP staining (Figure 4D and E). In contrast to the conditioned medium from C5a‐ and/or Pam3‐treated osteoblasts, the direct addition of C5a and Pam3 to the osteoclast medium did not induce osteoclastogenesis. Furthermore, the inhibition of C5aR1 and MyD88, simultaneously to the incubation with osteoblast‐conditioned medium, did not impair its osteoclastogenic potential (Figure 4D and E). To examine the osteoclastogenic effect of CXCL10 separately, recombinant CXCL10 was added to an additional treatment group, which showed strongly enhanced osteoclast formation, while this effect was reversed using a neutralizing CXCL10‐antibody (Figure 4D). Importantly, osteoclast formation mediated by the osteoblast supernatants was significantly attenuated when antagonizing CXCL10 (Figure 4D and E).

**Figure 5:**
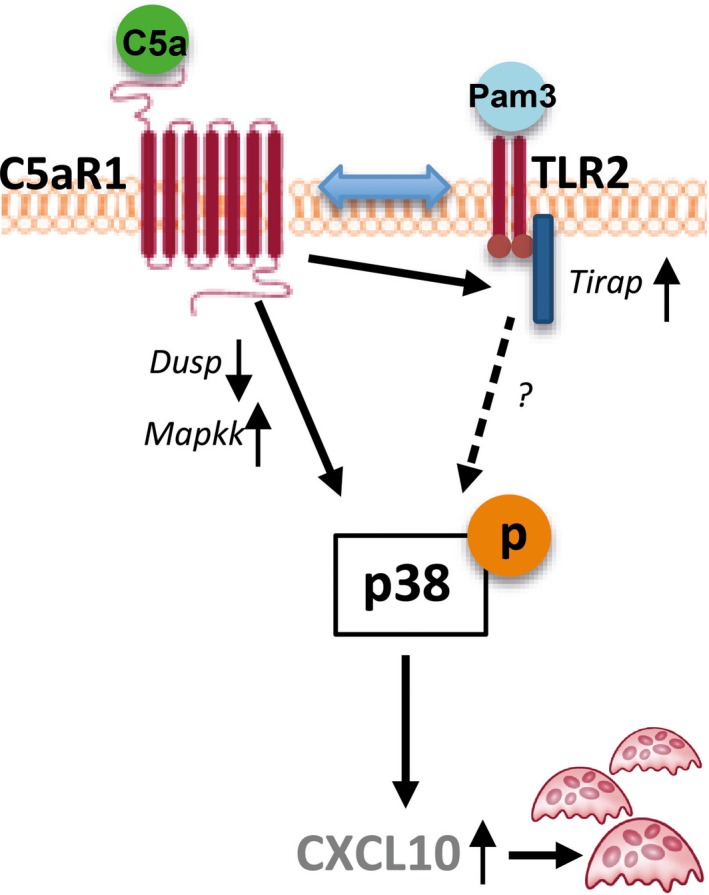
Current working model of C5aR1‐TLR2 interaction and crosstalk in downstream signalling. Pam3: Pam3CSK4, Dusp: dual specificity phosphatase, Mapkk: mitogen‐activated protein kinase kinase, Tirap: Toll‐interleukin 1 receptor (TIR) domain‐containing adaptor protein, CXCL10: C‐X‐C motif chemokine 10

The effects on osteoclast formation were confirmed on gene level, as genes encoding for TRAP (*Acp5*) and Cathepsin K (*Ctsk*) were induced by C5a‐ and Pam3‐treated osteoblast supernatant, and CXCL10 (data not shown). The findings of this study, regarding C5aR1 and TLR2 interactions in osteoblasts and their convergence in downstream signalling pathways, are illustrated in the current working model (Figure 5).

## 4 DISCUSSION

In this study, we demonstrated that C5a modulates the expression of genes involved in the MAPK and TGF‐β pathways, insulin and interferon signalling and the AP‐1 transcription factor in osteoblasts. We further showed that C5aR1 and TLR2 interact in osteoblasts and crosstalk in downstream signalling. The pathways converge on the activation of p38 MAPK, eventually leading to expression of the chemokine CXCL10. To unravel intracellular events following activation of the C5a/C5aR1 axis, we performed whole genome‐covering microarray analyses. Interestingly, we found a C5a‐mediated induction of genes related to insulin signalling and glucose metabolism. Negative insulin regulators were downregulated, while *Gfpt2* expression was upregulated. GFPT2 regulates glucose flux, metabolism, and utilization. It converts fructose‐6‐phosphate to glucosamine‐6‐phosphate and thereby catalyses the rate‐limiting step of the hexosamine biosynthesis pathway (HBP). High glucose flux into the HBP is associated with insulin resistance, impaired glucose tolerance and type 2 diabetes,^30^ effects which could be linked to increased GFPT2 activity.^31^, ^32^ Notably, C5a influences glucose metabolism in neutrophils, leading to increased glucose uptake and glycolysis, thus resembling insulin action in these cells.^33^, ^34^ Moreover, C5aR1 contributes to insulin resistance in an in vivo obesity model.^35^ These data imply that complement, and in particular the C5a/C5aR1 axis, might affect glucose metabolism not only in immune cells but also in osteoblasts. This might be the case particularly under high bone‐turnover conditions, requiring increased energy supply.^36^ Taken together, under inflammatory conditions, C5a might tilt the metabolic balance in osteoblasts, thereby affecting both bone turnover and local and systemic glucose homeostasis. To strengthen the link between complement and glucose metabolism, further investigation is required. C5a‐activated HBP may enhance proteoglycan production, which is important for bone structure and interacts with growth factors present in bone matrix, such as TGF‐β.^37^, ^38^ Interestingly, we found C5a‐induced genes to be involved in the TGF‐β pathway. TGF‐β is released from bone matrix during bone resorption, co‐ordinating bone formation and osteoblast activity. It promotes osteoblast differentiation 39 and migration of osteoblast precursor cells to the bone‐turnover site.^40^ TGF‐β can display pro‐osteoclastogenic actions, even in absence of RANKL,^41^ a finding supporting our hypothesis of a C5a‐mediated stimulation of osteoclastogenesis via osteoblasts. Of note, data on the effects of TGF‐β on osteoclasts are controversially discussed. Depending on the microenvironment and the maturation state of the cells, TGF‐β can also inhibit osteoclast formation and induce osteoclast apoptosis.^42^, ^43^ Interferon‐mediated actions are important to regulate the immune response to microbial and viral infections. Interferon‐γ (IFN‐γ) acts immunomodulatory, but also influences bone metabolism. IFN‐γ can act anabolic on bone and is able to rescue an osteoporotic phenotype.^44^ In addition, IFN‐γ strongly impacts osteoclastogenesis and RANKL signalling.^45^ Here, we found interferon signalling among the top C5a‐regulated pathways, suggesting an effect of C5a on interferons as regulators of both bone and the immune system. Furthermore, components of the AP‐1 transcription factor, namely JunB, c‐Jun and c‐Fos were strongly downregulated by C5a. Reduced AP‐1 activity was found in inflammatory skin disease and arthritis,^46^, ^47^ and therefore, downregulation of *Jun* and *Fos* observed here could possibly contribute to the proinflammatory cell phenotype of osteoblasts after C5a stimulation. In addition to characterizing the C5a/C5aR1 axis in isolation, we were interested in a possible crosstalk with other systems of innate immunity and demonstrated for the first time that the complement and TLR systems interact in bone cells. Both systems are important for early pathogen recognition, and their interplay allows sufficient co‐ordination of early immune responses. Beyond its function in host defence, complement regulates normal tissue homeostasis and regeneration processes, but also contributes to inflammatory diseases, alone or in combination with TLRs.^18^ The role of complement in bone development and regeneration after traumatic injury has been reviewed.^3^, ^4^ Fewer data are available on the impact of TLRs on bone, still, they have been ascribed a clear impact on inflammation and bone resorption in an infectious microenvironment, for example in periodontitis.^48^, ^49^ Of note, C5aR1 is also crucial for the development of periodontal disease, which is frequently accompanied by bone loss and subsequent tooth loss.^10^, ^11^, ^50^ Pharmacological inhibition of C5aR1 reduces periodontal inflammation and can reverse an already established disease.^11^ Crosstalk between TLR2 and C5aR1 promotes periodontal inflammation and concomitant bone loss,^10^, ^11^ and both receptors colocalize in macrophages challenged by the periodontitis keystone pathogen *Porphyromonas gingivalis* (*P. gingivalis*).^10^, ^51^ These data on C5aR1‐TLR2 interactions are mainly derived from immune cells and primarily suggest a modulation of the immune system. It remains unclear whether bone cells themselves can contribute to inflammatory bone conditions, including periodontitis, osteoarthritis and osteomyelitis. The involvement of bone cells might particularly hold true in a primarily locally restricted inflammation of the periodontium, where activated osteoblasts and osteoclasts could influence the inflammatory micromilieu. Therefore, a deeper understanding of osteoblast responses to inflammatory stimuli, mimicked here by the application of C5aR1 and TLR2 ligands, is key to managing inflammatory bone diseases in future. Notably, osteoblasts express both C5aR1 and TLR2,^5^, ^6^, ^52^ thus enabling these cells to react to complement activation and to recognize bacterial components. Here, we found a strong upregulation of TLR2 after osteogenic differentiation (Figure 3A–D), suggesting that TLR2 is crucially involved in osteoblast metabolism. Indeed, studies showed that osteoblasts produce the osteoclast‐stimulator RANKL in response to bacterial‐induced TLR2 activation.^48^, ^53^ Additionally, C5aR1‐mediated effects in osteoblasts involve the induction of RANKL.^5^ However, detailed molecular mechanisms underlying C5aR1 actions in osteoblasts remain unknown. We describe here that C5a activates p38 MAPK in osteoblasts, an intracellular effect also seen upon TLR2 activation using Pam3. Importantly, simultaneous stimulation of both receptors led to an additive effect thereon. Furthermore, genes involved in MAPK signalling were found to be regulated by C5a in the present study. MAPKs are important signalling molecules and known to be activated in a C5a‐dependent manner in neutrophils 54 and macrophages.^55^ This suggests similarities between immune cells and osteoblasts, not only in intracellular signalling but possibly also regarding inflammatory responses. Notably, p38 MAPK was found to be activated in inflammatory diseases associated with bone loss, including rheumatoid arthritis.^56^, ^57^ In a murine model of experimental arthritis, the pharmacological inhibition of p38 MAPK could reverse cartilage and bone destruction.^58^

Osteoblasts are increasingly regarded to act as proinflammatory cells, which produce cytokines, for example, in response to bacterial stimuli.^59^ In this study, we demonstrated that osteoblasts express CXCL10 in response to both C5a and Pam3. CXCL10 attracts mainly neutrophils, macrophages and cytotoxic T cells and is generated by many cell types, including osteoblasts, which express and secrete CXCL10 in response to bacterial challenges, namely by *Salmonella* 60 and *P. gingivalis*.^61^ Moreover, CXCL10 was found to act osteoclastogenic, either directly or indirectly by inducing RANKL expression by T cells and osteoblasts.^20^, ^21^ Furthermore, CXCL10 induction via RANKL was found to crucially contribute to bone destruction at inflamed joint areas during rheumatoid arthritis.^22^, ^62^ Here, we demonstrated that CXCL10 expression and secretion by osteoblasts was significantly enhanced under coactivation of C5aR1 and TLR2 compared to isolated receptor stimulation. Therefore, combined actions of C5aR1 and TLR2 in osteoblasts might modulate the immune system and promote osteoclastogenesis during inflammatory bone conditions. CXCL10 seems to be one out of several osteoclastogenic factors, which are induced by C5aR1‐TLR2 interaction, and CXCL10 could thereby act as an important coupling factor between osteoblasts and osteoclasts. Importantly, CXCR3, the receptor for CXCL10, is expressed by both osteoblasts and osteoclasts,^63^, ^64^ which we confirmed here on gene level. Therefore, osteoclasts are indeed potential target cells of osteoblast‐secreted CXCL10, which could in turn also activate osteoblasts in a feedback loop. The performed osteoclast formation assay did confirm the osteoclastic potential of both CXCL10 alone and C5a‐ and Pam3‐induced osteoblast‐secreted CXCL10. By using a neutralizing antibody against CXCL10, we showed that the osteoclastogenic effect mediated by the applied osteoblast conditioned media was significantly reduced, however, not completely abolished. These findings indicate that CXCL10 is a crucial, but probably not the only osteoclastogenic factor induced by C5aR1 and TLR2 activation.

A limitation of this study is that we focused on C5aR1‐mediated effects and did not entirely distinguish between the effects of the two receptors for C5a, C5aR1, and C5aR2. In a recent in vivo study, we demonstrated that the lack of C5aR1 or C5aR2 differentially affected bone cells and the early inflammatory phase of fracture healing.^8^ Therefore, further studies are required, dissecting C5aR1‐ from C5aR2‐mediated effects in osteoblasts, to enable tailored C5a receptor‐modulation under inflammatory bone conditions in future.

In summary, we demonstrated the interaction of C5aR1 and TLR2 in osteoblasts, not only physically but also functionally regarding downstream signalling and target genes, including the immune cell chemoattractant and osteoclastogenic mediator CXCL10. By inducing CXCL10, osteoblasts possibly contribute to inflammation and bone resorption during infectious conditions of the bone. Therefore, C5aR1, TLR2, and CXCL10 provide potential targets for therapeutic interventions in treating and controlling bone infections. Caution has to be exercised, however, when therapeutically manipulating C5aR1, as proper bone regeneration requires a tight control of receptor activity and a balance between its negative and positive effects on bone healing.^65^ Future in vivo investigations modelling infectious bone disease are required to corroborate the involvement and interaction of C5aR1 and TLR2 in bone inflammation, as suggested in this in vitro study.

## CONFLICT OF INTEREST

The authors confirm that there are no conflicts of interest.

## AUTHOR CONTRIBUTIONS

YM: research design, data acquisition, data analysis and interpretation, manuscript preparation; AR: data interpretation and revision of the manuscript; JP and KH: data analysis and interpretation; AK, MHL, and MHL: data interpretation, scientific discussions; AI: research design, data interpretation, manuscript preparation. All authors reviewed and approved the final version of the manuscript.
