# Supplementary figures and images for: C5aR1 interacts with TLR2 in osteoblasts and stimulates the osteoclast‐inducing chemokine CXCL10
Source: J Cell Mol Med. 2018 Sep 24;22(12):6002–14. doi: 10.1111/jcmm.13873 (PMC6237570; doi:10.1111/jcmm.13873)

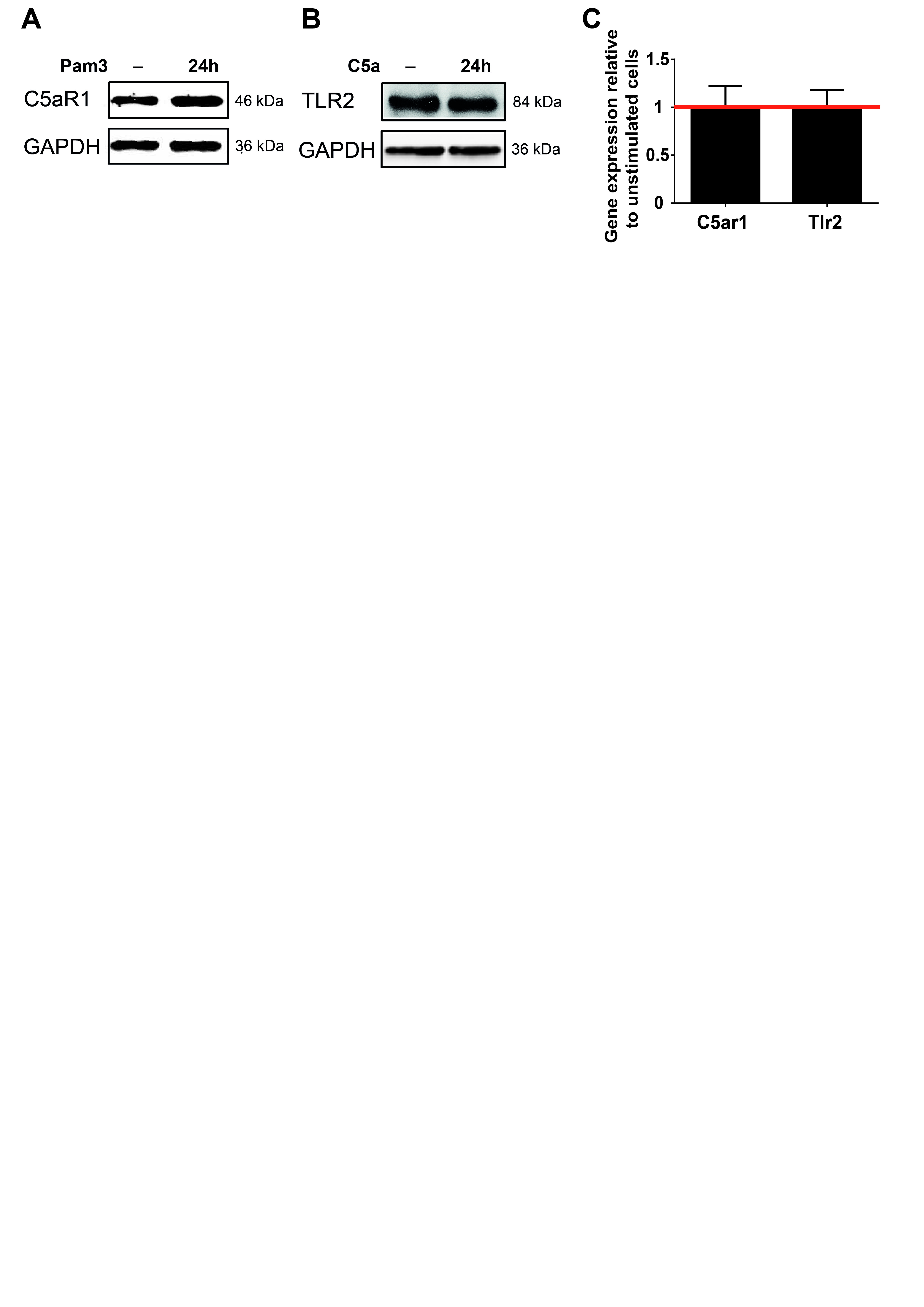

Supplement: Supplementary file 1 [file JCMM-22-6002-s001.jpg]

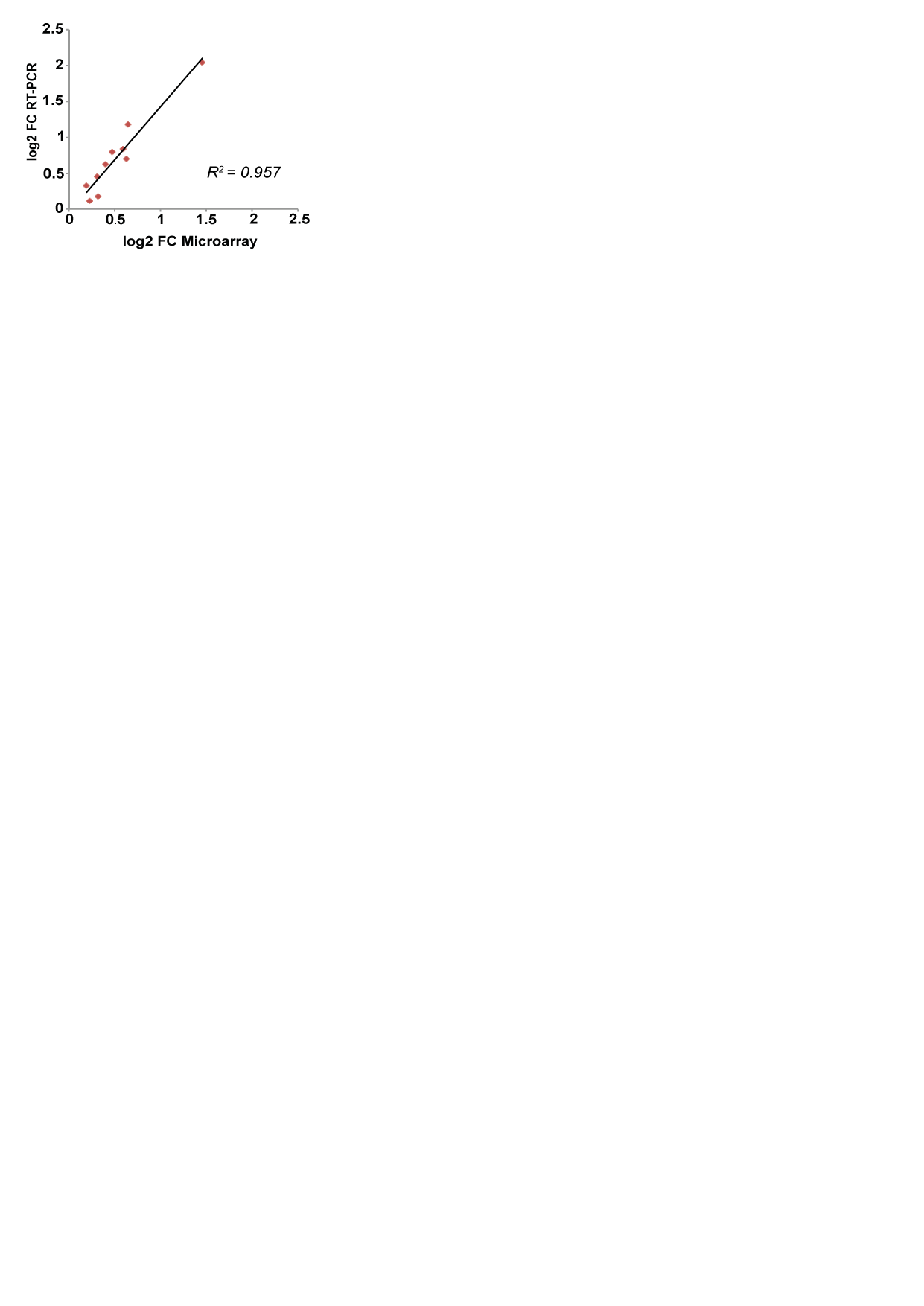

Supplement: Supplementary file 2 [file JCMM-22-6002-s002.tif]
