# Supplementary material for: C5aR1 interacts with TLR2 in osteoblasts and stimulates the osteoclast‐inducing chemokine CXCL10
Source: J Cell Mol Med. 2018 Sep 24;22(12):6002–14. doi: 10.1111/jcmm.13873 (PMC6237570; doi:10.1111/jcmm.13873)
Supplement: Supplementary file 3 [file JCMM-22-6002-s003.docx]

**Supporting Information**

**Tables**

**Table S1: Primer sequences**

| **Target Gene** | **Forward Primer Sequence (5'–3')** | **Reverse Primer Sequence (5'–3')** |
| --- | --- | --- |
| *Cxcl10* | GGA TCC CTC TCG CAA GGA | ATC GTG GCA ATG ATC TCAA CA |
| *C5ar1* | GGC CAT CCT GCG GCT GAT GG | GCC TTG CGA CTC CAG GTC CG |
| *Dusp1* | CTT CCG AGA AGC GTG ATA GG | TGC AGC TCC TGT AGT ACC CC |
| *Dusp5* | ACC CAG AAG ACT GTG GAT GG | GGA TGC AGG GAT GAT GTT CT |
| *Fos* | CCA GTC AAG AGC ATC AGC AA | ATG ATG CCG GAA ACA AGA AG |
| *Gapdh* | ACC CAG AAG ACT GTG GAT GG | GGA TGC AGG GAT GAT GTT CT |
| *Gfpt2* | ATG TGC GGA ATC TTT GCC TAC | AGA CCC CTG ATA AGG GTT TCG |
| *Grb14* | TAG AAA GAA CCG TAG AGG ACC AC | CAC CAT GTG CTC TGG AAA GAA |
| *Jun* | AAA ACC TTG AAA GCG CAA AA | GCT TAA GCT GTG CCA CCT GT |
| *Mapk6* | TGG GGC TGA AAG TCA GAA TC | AAC GAC ATG ACT GAG CCA CA |
| *Map2k3* | GGT GTG GGG TTG GAC ACA | CAG CTT GCC TCA GAC CAA A |
| *Socs3* | TGC GCC TCA AGA CCT TCA G | GCT CCA GTA GAA TCC GCT CTC |
| *Tgfbi* | ATT TGA GAG CGG AAG AGC TG | ACC AGA GGA AGA TCT GCG G |
| *Tgfbr1* | AGA CCA TCT GTC TCA CAG GTA AAA | CTC CTC ATC GTG TTG GTG G |
| *Tgfb1* | CAA CCC AGG TCC TTC CTA AA | GGA GAG CCC TGG ATA CCA AC |
| *Tirap* | CCA TGG CCT TCA ACA GTC TT | ACC AAG CCA CTT TTC ACA GG |
| *Tlr2* | AGA GGA CTG TTA TGG CCA CC | AGC ATC CGA ATT GCA TCA CC |

*Cxcl10: C-X-C motif chemokine 10, C5ar1: Complement component 5a receptor 1, Dusp1: Dual specificity phosphatase 1, Dusp5: Dual specificity phosphatase 5, Fos: Fos Proto-Oncogene, AP-1 Transcription Factor Subunit, Gapdh: Glyceraldehyde-3-phosphate dehydrogenase, Gfpt2: Glutamine-Fructose-6-Phosphate Transaminase 2, Grb14: Growth factor receptor-bound protein 14, Jun: Jun Proto-Oncogene, AP-1 Transcription Factor Subunit, Mapk6: Mitogen-activated protein kinase 6, Map2k3: Mitogen-activated protein kinase kinase 3, Socs3: Suppressor of cytokine signaling 3, Tgfbi: Transforming growth factor beta induced, Tgfbr1: Transforming growth factor beta receptor 1, Tgfb1: Transforming growth factor beta, Tirap: Toll-interleukin 1 receptor (TIR) domain-containing adaptor protein, Tlr2: Toll-like receptor 2.*

**Table S2. Top 20 regulated pathways upon C5a treatment in osteoblasts.**

| **Order** | **Pathway** | **Genes Up** | **Genes Down** | **Significance** | **p-value** |
| --- | --- | --- | --- | --- | --- |
| 1 | Insulin signaling | 8 | 12 | 9.48 | <1×10^-6^ |
| 2 | TGF-beta receptor signaling | 9 | 8 | 7.47 | <1×10^-6^ |
| 3 | TGF-beta signaling | 6 | 4 | 6.8 | <1×10^-6^ |
| 4 | MAPK signaling | 6 | 10 | 6.39 | <1×10^-6^ |
| 5 | MAPK signaling | 6 | 10 | 5.99 | 1×10^-6^ |
| 6 | ESC pluripotency pathways | 7 | 6 | 5.72 | 2×10^-6^ |
| 7 | Adipogenesis genes | 5 | 8 | 5.14 | 7×10^-6^ |
| 8 | Type II interferon signaling | 0 | 7 | 5.14 | 7×10^-6^ |
| 9 | Spinal cord injury | 5 | 6 | 4.88 | 1×10^-5^ |
| 10 | Endochondral ossification | 6 | 2 | 4.23 | 6×10^-5^ |
| 11 | p38 MAPK signaling | 2 | 4 | 4.08 | 8×10^-5^ |
| 12 | Podocyte protein interactions | 24 | 11 | 3.57 | 3×10^-4^ |
| 13 | Il-6 signaling pathway | 1 | 8 | 3.5 | 3×10^-4^ |
| 14 | Oxidative stress | 3 | 2 | 3.4 | 4×10^-4^ |
| 15 | EGFR1 signaling pathway | 4 | 8 | 3.29 | 5×10^-4^ |
| 16 | Toll-like receptor signaling | 2 | 6 | 2.89 | 1×10^-3^ |
| 17 | IL-2 signaling pathway | 2 | 5 | 2.87 | 1×10^-3^ |
| 18 | IL-3 signaling pathway | 4 | 4 | 2.8 | 2×10^-3^ |
| 19 | Regulation of actin cytoskeleton | 6 | 4 | 2.74 | 2×10^-3^ |
| 20 | B cell receptor signaling | 4 | 6 | 2.64 | 2×10^-3^ |

**Table S3. Top 20 upregulated probesets upon C5a treatment in osteoblasts, representing respective genes**

| **Gene** | **Description** | **GenBank*** | **p-value** | **FC MA^#^** |
| --- | --- | --- | --- | --- |
| *Ngf*  *Itga5* | Nerve growth factor  Integrin alpha 5 | NM_001112698  NM_010577 | 3.3×10^-3^  2.3×10^-3^ | 7.0 |
|  |  |  |  | 5.4 |
| *Tgfbi* | Transforming growth factor beta induced | NM_009369 | 3.4×10^-3^ | 4.9 |
| *Gm7265* | Predicted gene 7265 | AK087684 | 7.1×10^-3^ | 4.5 |
| *Inhba* | Inhibin beta-A | NM_008380 | 1.3×10^-2^ | 4.5 |
| *Gfpt2* | Glutamine fructose-6-phosphate transaminase 2 | NM_013529 | 1.9×10^-3^ | 4.3 |
| *Nfatc2* | Nuclear factor of activated T cells 2 | NM_001037177 | 9.4×10^-3^ | 4.2 |
| *Mical2* | Microtubule associated monooxygenase 2 | NM_001193305 | 1.2×10^-2^ | 4.1 |
| *Il1rl1* | Interleukin 1 receptor-like 1 | NM_001025602 | 1.8×10^-2^ | 4.1 |
| *Pmepa1* | Prostate transmembrane protein 1 | NM_022995 | 2.8×10^-2^ | 4.1 |
| *Flnc* | Filamin C, gamma | NM_001081185 | 1.3×10^-2^ | 3.9 |
| *Chst11* | Carbohydrate sulfotransferase 11 | NM_021439 | 6.6×10^-3^ | 3.9 |
| *Gjb4* | Gap junction protein, beta 4 | NM_008127 | 3.5×10^-2^ | 3.9 |
| *Gjb3* | Gap junction protein, beta 3 | NM_001160012 | 2.2×10^-2^ | 3.7 |
| *Gprc5a* | G protein-coupled receptor, family C, 5A | NM_181444 | 4.8×10^-4^ | 3.6 |
| *Clcf1* | Cardiotrophin-like cytokine factor 1 | AK166218 | 1.1×10^-2^ | 3.5 |
| *Srxn1* | Sulfiredoxin 1 homolog | NM_029688 | 9.4×10^-3^ | 3.5 |
| *Txnrd1* | Thioredoxin reductase 1 | NM_001041513 | 1.4×10^-3^ | 3.5 |
| *Cspg4* | Chondroitin sulfate proteoglycan 4 | NM_139001 | 4.9×10^-3^ | 3.3 |
| *Ttc9* | Tertatricopeptide repeat domain 9 | NM_001033149 | 1.1×10^-2^ | 3.2 |

**Table S4. Top 20 downregulated probesets upon C5a treatment in osteoblasts, representing respective genes**

| **Gene** | **Description** | **GenBank*** | **p-value** | **FC MA^#^** |
| --- | --- | --- | --- | --- |
| *Fos* | FBJ osteosarcoma oncogene | NM_010234 | 1×10^-5^ | 28.6 |
| *Nr4a1* | Nuclear receptor subfamily 4, a1 | NM_010444 | 7.5×10^-3^ | 14.5 |
| *Ier2* | Immediate early response 2 | NM_010499 | 3.4×10^-3^ | 13.9 |
| *Fosb* | FBJ osteosarcoma oncogene B | NM_008036 | 5.2×10^-3^ | 12.4 |
| *Egr2* | Early growth response 2 | NM_010118 | 2.1×10^-4^ | 10.6 |
| *Zfp36* | Zink finger protein 36 | NM_011756 | 1×10^-4^ | 9.1 |
| *Btg2* | B cell translocation gene 2 | NM_007570 | 1.6×10^-4^ | 9.1 |
| *Kcne4* | Potassium voltage-gated channel 4 | NM_021342 | 1×10^-2^ | 6.7 |
| *Hpgd* | Hydroxyprostaglandin dehydrogenase 15 | NM_008278 | 3×10^-2^ | 6.2 |
| *Adamts1* | A disintegrin-like and metallopeptidase with trombospondin type 1, motif 1 | NM_009621 | 1.5×10^-2^ | 5.3 |
| *Mir145a* | microRNA 145a | NR_029557 | 1×10^-2^ | 4.6 |
| *Dusp1* | Dual specificity phosphatase 1 | NM_013642 | 1.9×10^-2^ | 4.4 |
| *Ier3* | Immediate early response 3 | NM_133662 | 1.1×10^-2^ | 4.2 |
| *Junb* | Jun B proto-oncogene | NM_008416 | 2.4×10^-3^ | 4.0 |
| *Dusp5* | Dual specificity phosphatase 5 | NM_001085390 | 8.4×10^-3^ | 3.6 |
| *Otud1* | OUT domain containing 1 | NM_027715 | 7.6×10^-3^ | 3.7 |
| *Egr1* | Early growth response 1 | NM_007913 | 8.9×10^-3^ | 3.6 |
| *Dnajb1* | Dnaj (Hsp40) homolog, B1 | NM_018808 | 1.9×10^-3^ | 3.5 |
| *Gpr88* | G-protein coupled receptor 88 | NM_022427 | 5×10^-3^ | 3.5 |
| *Dbp* | D site albumin promoter binding protein | NM_016974 | 1.4×10^-3^ | 3.5 |

**Figure legends**

***Fig. S1: C5aR1 and TLR2 expression upon reciprocal receptor stimulation.***

*C5aR1 (****A****) and TLR2 (****B****) protein expression in unstimulated and 24 h-stimulated osteoblasts with Pam3 (****A****) or C5a* *(****B****). C5ar1 and Tlr2 gene expression of 6 h-stimulated osteoblasts with Pam3 and C5a (****C****). Pam3: Pam3CSK4.*

***Fig. S2. Correlation between microarray and RT-PCR analyses.***

*Correlation between microarray and RT-PCR analyses* *regarding the logarithmic fold change (FC) of gene expression values derived from comparison of unstimulated with C5a-stimulated cells. RT-PCR: Reverse-transcription PCR.*
